# Efficacy of palliative radiotherapy for gastric bleeding in patients with unresectable advanced gastric cancer: a retrospective cohort study

**DOI:** 10.1186/s12904-015-0034-y

**Published:** 2015-08-04

**Authors:** Chihiro Kondoh, Kohei Shitara, Motoo Nomura, Daisuke Takahari, Takashi Ura, Hiroyuki Tachibana, Natsuo Tomita, Takeshi Kodaira, Kei Muro

**Affiliations:** Department of Clinical Oncology, Aichi Cancer Center Hospital, 1-1 Kanoko-den Chikusa-ku, Nagoya City, Aichi Japan; Department of Radiation Oncology, Aichi Cancer Center Hospital, 1-1 Kanoko-den Chikusa-ku, Nagoya City, Aichi Japan; Department of Medical Oncology, Japanese Red Cross Nagoya Daiichi Hospital, 3-35 Michishita-Cho, Nakamura-ku, Nagoya City, Aichi Japan; Department of Gastroenterology and Gastrointestinal Oncology, National Cancer Center Hospital East, 6-5-1 Kashiwanoha, Kashiwa City, Chiba Japan

**Keywords:** Gastric cancer, Palliative care, Radiotherapy, Hemostasis, Bleeding

## Abstract

**Background:**

Bleeding negatively impacts quality of life in patients with unresectable advanced gastric cancer and has the potential to be lethal. When blood transfusion and endoscopic hemostasis are unsuccessful to stop bleeding, radiation to stomach is selected in patients with unsuitable condition for surgery. We performed a retrospective cohort study to clarify the utility of radiotherapy in treating gastric bleeding, particularly for patients with limited life expectancy.

**Methods:**

We evaluated the efficacy and safety of palliative radiotherapy in patients with advanced gastric cancer between January 2007 and December 2012 in Aichi Cancer Center Hospital. All patients had gastric bleeding requiring blood transfusion. We defined hemostasis as an increase in hemoglobin level to more than 7.0 g/dL together with the cessation of melena or hematemesis for at least 1 week.

**Results:**

During the study period, 313 advanced gastric cancer patients treated in our institution. Of these 17 patients received gastric radiotherapy to stop bleeding. Two patients were excluded from analysis due to combined treatment of intravascular embolization. Eleven out of 15 patients (73 %) had undergone two or more previous chemotherapy regimens. Ten patients (67 %) had an Eastern Cooperative Oncology Group performance status of 3 and 14 patients (93 %) were in palliative prognostic index group B or C. The median total planned radiation dose was 30 Gy in 10 fractions. At a median interval of 2 days after initiation of radiotherapy, 11 patients (73 %) achieved hemostasis; rebleeding was observed in four patients (36 %). The median hemoglobin level before radiotherapy was significantly increased from 6.0 to 9.0 g/dL (*p* < 0.0001). The median volume of red blood cell transfusion was significantly decreased from 1120 to 280 mL (*p* = 0.007). The median rebleeding-free survival interval was 27 days, with a median overall survival of 63 days. The cause of death was bleeding in 1 patient (7 %) and cancer progression without bleeding in 12 patients (80 %). There were no severe adverse events attributable to radiotherapy.

**Conclusions:**

Palliative radiotherapy for gastric bleeding achieves hemostasis within a short time frame. This appears to be a useful treatment option, especially for patients with end-stage, unresectable advanced gastric cancer.

## Background

Adenocarcinoma of the stomach is a significant malady, with a worldwide incidence of more than 900 000, causing 700 000 deaths in 2012 [1]. Patients with early gastric cancer are commonly treated surgically and with advanced locoregional disease are treated with multimodal approach such as surgery followed by chemotherapy or chemoradiotherapy [2–4] or perioperative chemotherapy or chemoradiotherapy [5, 6]. For patients with advanced gastric cancer (AGC), quality of life is negatively impacted when the tumor causes obstruction and symptoms such as pain or bleeding. Hemoglobin level of less than 6.5 g/dL is categorized as life-threatning, blood transfusion is the only intervention option for patients who require immediate correction of anemia. According to the guidelines published by the American Association of Blood Banks, transfusion is recommended for hospitalized patients without cardiovascular disease targeting the threshold of hemoglobin level of 7 to 8 g/dL and depending on their symptoms. [7] Radiotherapy (RT) is sometimes used to treat severe anemia due to gastric bleeding in patients with AGC who are not able to undergo surgery, endoscopy, or intravascular embolization. The efficacy of RT for bleeding has been evaluated in patients with non-small-cell lung cancer, cervical cancer, and bladder cancer [8–11]. In patients with AGC, between 54 and 80 % are able to achieve hemostasis with RT [12–17]. In patients who complete a dose of 30 Gy or more, 91 % are hemostatic within 1 month [16]. The survival time of patients with AGC who fail standard chemotherapy is reportedly 3.8–4.3 months [18–20]. In the present study, we attempted to refine these measurements, focusing on the interval to achieve hemostasis after initiating RT and the eventual cause of death in patients with AGC. These parameters may be more important to patients who are seeking palliative rather than curative treatment.

## Methods

### Patients

For this retrospective cohort study, we reviewed the records of patients with unresectable AGC who received palliative RT to control gastric bleeding. All patients were treated at Aichi Cancer Center Hospital between January 2007 and December 2012. The principal inclusion criteria were: histologically proven inoperable adenocarcinoma in the stomach or in the esophagogastric junction, endoscopically confirmed gastric bleeding, and at least one blood transfusion administered to improve anemia before RT.

A total of 313 patients with AGC were treated at our institution during the study period. Of these, 17 patients had received gastric RT for hemostasis. Two patients with hemorrhagic shock were excluded from analysis due to a history of interventional radiology (IVR) procedures administered within 7 days of beginning RT. All patients gave written informed consent for treatment. The review board of Aichi Cancer Center approved this study and deemed informed consent unnecessary due to its retrospective nature.

### Radiotherapy

RT planning was performed using 3-dimensional radiation planning system with 5 or 10-mm slice thickness of computed tomography (CT) images. The clinical target volume (CTV) was defined as primary lesion, with adequate margins, based on the findings of both endoscopic examination and CT images. Regional lymph nodes were not included in the CTV. The planning treatment volume (PTV) is created from CTV adding margin for clinical treatment set-up. All patients were treated using external 10-MV photon beams from a linear accelerator. Typically, a total dose of 30Gy in 10 fractions was delivered to the PTV using the antero-posterior parallel opposed portals.

### Evaluation of treatment and statistical analysis

The Eastern Cooperative Oncology Group performance status (PS) and the Palliative Prognostic Index (PPI) were used to evaluate patients [21, 22]. PPI is calculated for each case based on palliative performance scale, the amount of oral intake, and the presence of edema, dyspnea at rest, and delirium. Patients are divided into groups according to projected survival time: group A, 4 to 5 months; group B, 3 months; and group C, less than 3 weeks [22].

Patients’ records were reviewed from the time point 30 days before initiating RT until their death. The day of hemostasis was defined without endoscopic evidence as the first day after starting RT that satisfied all of the following criteria for at least 7 consecutive days: an increase in hemoglobin to greater than 7.0 g/dL, no evidence of melena or hematemesis, and no indication for blood transfusion. Patients who required other hemostatic procedures such as IVR techniques or endoscopic procedures were categorized as failing RT. Patients given blood transfusions after achieving hemostasis were categorized as rebleeding.

Hematologic studies were conducted at least once weekly. The change in hemoglobin level was determined by evaluating the lowest point in the 30 days before starting irradiation and the level 30 days after starting RT; t-testing was used to compare these values. Toxicity was evaluated within 30 days of initiating RT using the National Cancer Institute Common Toxicity Criteria, version 4.0.

Biologically effective dose (BED) is an approximate quantity to compare the different fractionation regimens. Using alpha/beta ratio of 10 for adenocarcinomas, BED was calculated for each cases.

Rebleeding-free survival (RFS) was measured from the first day of RT until either rebleeding occurred or death from any cause took place. Rebleeding was set at day1 of RT for patients who never achieved hemostasis. Overall survival (OS) was measured from the first day of RT until death occurred. The median RFS and OS were determined using the Kaplan-Meier method. Statistical analysis was performed using JMP software, version 10.0.2 (Statistical Analysis Software, Inc., Cary, NC). Statistical significance was set at 0.05 (using 2-tailed testing).

## Results

### Patient characteristics

Patient characteristics are listed in Table 1. The median follow-up time was 35.4 months (range, 0.9–82.0 months). The median patient age was 61 years. Male patients predominated, and 67 % of all patients had a poor PS. A total of eight patients (53 %) were in PPI group B, and six patients (40 %) were in group C. The dominant histology was poorly differentiated adenocarcinoma in seven patients (47 %), and the tumor was located in the gastric body in 11 patients (73 %). All patients had metastatic disease, and 10 (67 %) had organ metastases. Eleven patients (73 %) had received two or more chemotherapy regimens prior to beginning RT (median, 3 regimens).

**Table 1:** Patient characteristics

Characteristics		Radiotherapy	Chemoradiotherapy^b^	Total
		(*n* = 10) % (*n*)	(*n* = 5) % (*n*)	(*n* = 15) % (*n*)
Age (years)				
	Median (range)	62 (57–87)	53 (42–71)	61 (42–87)
Sex				
	Male	70 (7)	80 (4)	73 (11)
ECOG performance status				
	1	0	20 (1)	1 (7)
	2	30 (3)	20 (1)	4 (26)
	3	70 (7)	60 (3)	10 (67)
Palliative prognostic index group [22]				
	A	0	20 (1)	7 (1)
	B	70 (7)	20 (1)	53 (8)
	C	30 (3)	60 (3)	40 (6)
Histological type				
	Well differentiated	10 (1)	20 (1)	13 (2)
	Moderately differentiated	50 (5)	20 (1)	40 (6)
	Poorly differentiated	40 (4)	60 (3)	47 (7)
Location				
	Esophagogastric junction	20 (2)	40 (2)	27 (4)
	Gastric corpus	80 (8)	60 (3)	73 (11)
Metastatic site^a^				
	Lymph nodes	60 (6)	80 (4)	67 (10)
	Liver	60 (6)	60 (3)	60 (9)
	Peritoneum	30 (3)	60 (3)	40 (6)
Prior chemotherapeutic regimen				
	0	10 (1)	0	7 (1)
	1	20 (2)	20 (1)	20 (3)
	2	10 (1)	20 (1)	13 (2)
	3 or more	60 (6)	60 (3)	60 (9)
Subsequent chemotherapy^c^				
	Yes	20 (2)	60 (3)	33 (5)
Radiotherapy dose (Gy)				
	30	80 (8)	80 (4)	80 (12)
	>30	10 (1)	20 (1)	13 (2)
	<30	10 (1)	0	7 (1)
Biologically effective dose (Gy_10_)				
	Median (range)	39 (23.4–43.2)	39 (39–48)	39 (23–48)

*ECOG* Eastern Cooperative Oncology Group

^a^Overlapped data

^b^Paclitaxel in 1; paclitaxel and trastuzumab in 1; methotrexate + 5-fluorouracil (5-FU) in 1; oxaliplatin + folinic acid + 5-FU in 1; low dose cisplatin + 5-FU in 1

^c^Paclitaxel in 2; methotrexate + 5-FU in 1; docetaxel in 1; trastuzumab + lapatinib in 1

Concurrent chemotherapy was administered in five patients (33 %). The regimens were as follows: weekly paclitaxel, weekly paclitaxel and trasutuzumab, methotrexate and 5-fluorouracil (5-FU), low-dose cisplatin and 5-FU, and FOLFOX (oxaliplatin, folinic acid and 5-FU). Compared with the concurrent chemoRT group, the RT alone group was older in median age. PPI group C and peritoneal metastasis were less in RT alone group. The other background factors were balanced between the two groups. Chemotherapy regimens after completion of RT were as follows: weekly paclitaxel in two patients, methotrexate and 5-FU in one patient, trastuzumab and lapatinib in one patient, and docetaxel in one patient. Subsequent chemotherapy was administrated to two patients (20 %) in RT alone group and to three patients (60 %) in chemoRT group.

### Treatment results

Twelve patients (80 %) received the dose-fraction regimen of 30 Gy in 10 fractions corresponding to BED 39 Gy_10_. The others were planned as 40Gy in 20 fractions, 36Gy in 18 fractions, and 30Gy in 12 fractions for one patient respectively; these doses were completed in all but a single patient (93 %) (Table 1). One patient with brain metastases discontinued RT after achieving hemostasis because of restlessness during irradiation. All patients were treated with supportive medications such as proton pump inhibitors, tranexam acid and local thrombin.

**Fig. 1:**
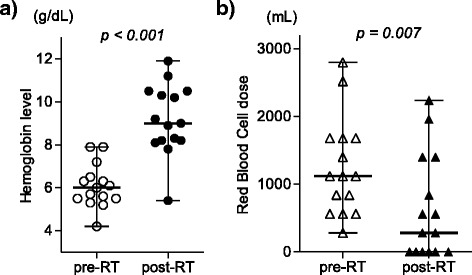
**a** Comparison of hemoglobin levels prior to and 30 days after initiation of gastric radiation therapy (RT). The horizontal bars represent the mean (long bar) and the range (short bars). **b** Comparison of the amount of transfused red blood cells 30 days before and 30 days after starting gastric RT. The horizontal bars represent the mean (long bar) and the range (short bars)

**Fig. 2:**
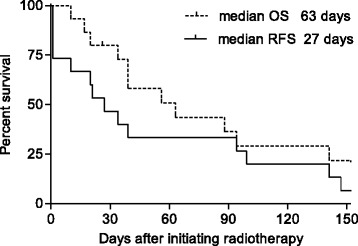
Rebleeding-free survival (RFS) and overall survival (OS) after gastric radiation therapy

Eleven patients (73 %) achieved hemostasis. The median time to hemostasis was 2 days (range, 1–9 days). The median hemostatic radiation dose was 6 Gy (range, 3–21 Gy). In the four patients categorized as no hemostasis, no one started RT with thrombocytopenia. Three patients received RT alone and one patient received chemoRT. One patient was rescued by arterial embolization technique and stopped bleeding. None was performed endoscopic procedures. The median hemoglobin level before RT was 6.0 g/dL; after 30 days, the median was 9.0 g/dL, a significant increase (*p* < 0.001). The median platelet count was 291,000/mm^3^ (range, 6.0–53.3) on the day of starting RT. The median red blood cell transfusion volume per month significantly decreased, from 1120 mL pre-RT to 280 mL after starting RT (*p* = 0.007) (Fig. 1). In the 11 patients who achieved hemostasis, 4 experienced rebleeding (36 %). At the time of rebleeding, the platelet count less than 50,000/mm^3^ was observed in one patient with disseminated intravascular coagulopathy due to bone marrow metastasis. The median RFS was 27 days (95 % confidential interval [CI], 1–94 days) (Fig. 2).

The cause of death was gastric bleeding in one patient (7 %) and cancer progression in 12 patients (80 %). Two patients (13 %) were still alive at the end of the study period. The median OS was 63 days (95 % CI, 20–141 days) (Fig. 2).

In the five patients of chemoRT goup, four patients stopped bleeding. Of them, two patients showed rebleeding 27 and 147 days later respectively. The median OS was 63 days (95%CI, 39–259 days).

### Toxicity

Grade 1 nausea and pleural effusion were documented in two patients. Fatigue, vomiting, and diarrhea were noted in one patient each. One patient required treatment for grade 2 hypertension after achieving hemostasis. As a result of concurrent or subsequent chemotherapy, Grade 3/4 neutropenia was documented in 3 patients. Grade 4 thrombocytopenia and creatinine elevation developed in one patient each, findings we attributed to disease progression (Table 2).

**Table 2:** Treatment toxicity

Toxicity	Grade			
	1	2	3	4
Nausea	2	0	0	0
Vomiting	1	0	0	0
Diarrhea	1	0	0	0
Fatigue	1	0	0	0
Pleural effusion	2	0	0	0
Hypertension	0	1	0	0
Creatinine increased	1	0	0	1
Bilirubin increased	2	1	0	0
Neutropenia	3	0	1	2
Thrombocytopenia	2	1	0	1

## Discussion

Gastric cancer is not adequately sensitive to RT alone; thus, multimodal therapeutic approaches with surgery and chemotherapy have been developed [2–6, 23]. Henning et al. showed an advantage in locoregional control in patients undergoing chemoradiation who received a dose of greater than 54 Gy, but no OS benefit was seen [24]. However, from the palliative point of view, Asakura et al. reported that a radiation dose of 30 Gy in ten fractures is adequate to control bleeding from gastric cancer [15]. The hemostatic rate observed in the present study is almost the same as Asakura et al’s and supports their results.

The mechanism of hemostasis induced by irradiation is not clearly identified. The irradiation procedure is widely thought to aggregate platelets or to damage the vascular endothelial cells. Radiation has also been shown to induce embolism of vessels, both *in vitro* and *in vivo* [25–27]. In the preclinical experimental models of rats and mice, platelet aggregation is observed 3 min after irradiation [28], with tissue factor (the primary initiator of blood coagulation, expressed on peripheral mononuclear cells) appearing after 1 day. Procoagulant activity is seen for duration of 7 days [29]. These mechanisms may support the early hemostatic response to RT observed in our patients.

OS and RFS are strongly associated with individual patient characteristics. Previous reports of palliative RT performed for gastric bleeding had study populations of 30–40 % chemo-naïve patients; 60–80 % had a good PS (PS1/2). Our study population was 7 % chemo- naïve, and 33 % had a good PS; hence, the prognosis of our patients is the poorest reported thus far. Fourteen of our patients (93 %) were categorized as PPI group B or C, a finding that confers a prognosis of less than 3 months survival but also indicates potential benefit from the use of RT. In patients with such a limited life expectancy, it is important to use minimally invasive treatment methods whenever possible. Hypofractionated RT was previously investigated, in a randomized, controlled trial, for the management of patients with bladder cancer who are unsuitable for curative treatment and who have an estimated 3-month survival prognosis [11]. A dose of 21 Gy in 3 fractions, given on alternate weekdays over 1 week, and a dose of 35 Gy in 10 fractions, given over 2 weeks, produced improvement in symptoms in 64 and 71 % of patients, respectively, with no evidence of a difference in efficacy or toxicity between the dosage groups. Hypofractionated RT may induce more late toxicity in patients with other types of cancer [30], so it is recommended only for patients with a limited life expectancy. In the setting of AGC refractory to standard chemotherapy, hypofractionated RT might be an option for patients in PPI group B or C.

Previous studies have not provided information about the cause of death in their patients. Although our results may be fairly premature, with two patients still living, 80 % of our patients died from disease progression, exhibiting organ failure or physical debilitation, not bleeding from stomach.

One of the major limitations of the present study is the method of patient selection. We excluded two patients from analysis because they had previously undergone intravascular catheter embolization. One of these patients achieved hemostasis with RT alone, but the other patient never stopped bleeding and went on to die of hemorrhage. One of the study patients had continuing hemorrhage after starting RT alone and underwent IVR rescue treatment for a pseudoaneurysm, detected along a branch of the left gastric artery. The condition common to these 3 patients is hemorrhagic shock, which may be an indicator of unsuitability for RT. Other limitations of this study are the small sample size and the retrospective, single-institution study design.

## Conclusions

In conclusion, our results, although limited, suggest that palliative RT may be a useful treatment option to control gastric bleeding in patients with unresectable AGC. Patients who respond to treatment typically achieve hemostasis within 2 days, and the benefits persist for longer than 1 month. These patients may avoid death from hemorrhage. Additional investigation is necessary in order to clarify the ideal RT dose and to select the most appropriate candidates for treatment.

## Abbreviations

AGC: Advanced gastric cancer
RT: Radiotherapy
IVR: Interventional radiology
CT: Computed tomography
CTV: Clinical target volume
PTV: Planning treatment volume
PS: Performance status
PPI: Palliative prognostic index
BED: Biologically effective dose
RFS: Rebleeding-free survival
OS: Overall survival
5-FU: 5-fluorouracil
CI: Confidential interval
